# Back-Gated WS_2_‑Enhanced Barium Titanate Phototransistor with Polarization-Controlled UV Responsivity and Enhanced Thermal Stability

**DOI:** 10.1021/acsami.5c15016

**Published:** 2025-11-19

**Authors:** Rohit Raj Padhi, Chiranjit Das, Guo-Hua Feng

**Affiliations:** † Institute of NanoEngineering and Microsystems, 34881 National Tsing Hua University, Hsinchu 30013, Taiwan; ‡ Department of Power Mechanical Engineering, National Tsing Hua University, Hsinchu 30013, Taiwan

**Keywords:** UV photodetector, ferroelectric field-effect transistor (FeFET), barium titanate (BTO), tungsten disulfide (WS_2_), remanent polarization, responsivity, switching ratio

## Abstract

A high-performance ultraviolet (UV) phototransistor was developed utilizing a WS_2_/barium titanate (BTO) heterostructure within a back-gated ferroelectric field-effect configuration. BTO nanoparticles, prepared via hydrothermal synthesis, were spin-coated onto a TiO_2_ layer, while monolayer WS_2_ was drop-cast between aluminum source/drain electrodes. The intrinsic ferroelectric field, approximately 60 kV/cm, facilitated polarization-induced carrier modulation, resulting in a low dark current of 12.3 pA without the need for an external gate bias. Ultraviolet-visible (UV–vis) spectroscopy confirmed a bandgap reduction from 3.32 eV for pure BTO to 3.13 eV for the WS_2_/BTO composite, enhancing UV–vis light absorption. Raman spectroscopy further affirmed the structural integrity of WS_2_ postintegration. The integration of WS_2_ led to a decrease in the coercive field from 40 to 20 kV/cm, and a reduction in remanent polarization from 2.5 to 2.0 μC/cm^2^, indicating more efficient polarization switching. Photocurrent measurements under cyclic poling demonstrated a significant 28-fold enhancement, with the current increasing from 0.8 to 22.5 μA, and a peak responsivity of 38.5 A/W was achieved. The device exhibited a rapid rise time of 0.8 s, maintained a switching ratio exceeding 10^4^, and demonstrated stable operation across a temperature range of 40–80 °C. These findings indicate the strong promise of WS_2_/BTO heterostructures for energy-efficient, polarization-modulated UV photodetection, suitable for applications in memory devices, optical sensors, and wearable electronics.

## Graphical abstract

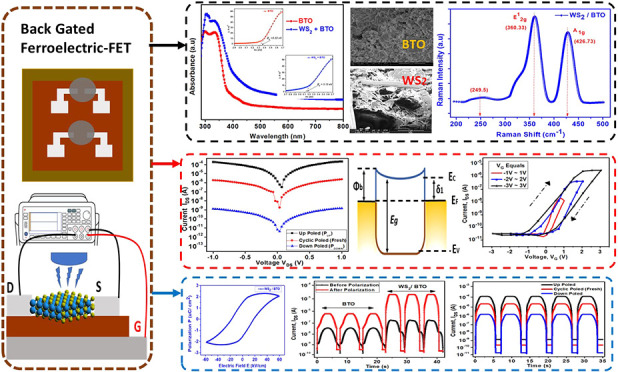

## 1 Introduction

The rising demand for advanced ultraviolet (UV) photodetectors is driven by critical applications in environmental monitoring, healthcare, and safety.^1–3^ As integrated circuit technology progresses, there is a pressing need for sensitive, reliable, and energy-efficient photodetectors.^4^ Two-dimensional (2D) materials have emerged as promising candidates due to their high carrier mobility, adjustable band gaps, and ultrathin structures.^5,6^ Nevertheless, their practical implementation faces notable challenges. The high surface-to-volume ratio of these materials often leads to inefficient heat dissipation, while their inherently low optical absorption requires stronger illumination to achieve effective photoresponse. Furthermore, their need for high external voltages hampers integration into energy-efficient systems.^7–9^ These constraints highlight the need for creative designs, such as the use of ferroelectric field-effect transistors (FeFETs) and sophisticated materials, to improve the sensitivity, stability, and overall performance of 2D phototransistors in UV detection applications.^10–12^

To overcome the challenges associated with two-dimensional (2D) material-based phototransistors, the integration of ferroelectric materials presents a compelling solution. Ferroelectrics possess inherent spontaneous polarization, which generates strong local electric fields that improve charge carrier mobilization, address heat management issues, and enhance stability under thermal fluctuations.^13,14^ Additionally, the negative capacitance effect of ferroelectric materials significantly boosts sensitivity and optical absorption, effectively compensating for the low-absorption cross-section of 2D materials and enabling effective operation under lower light intensities. This integration reduces the reliance on large external voltages, aligns with energy-efficient systems, and ensures reliable performance due to its nonvolatile characteristics.^15–18^

Given these advantages, modifying the gate dielectric in 2D transistors is crucial for several reasons. First, it enhances electrostatic control over the channel, optimizing carrier concentration and improving overall device performance, which can be useful for MOSFET or FeFET optimization.^19,20^ Second, the negative capacitance effect offered by ferroelectric materials leads to voltage amplification, which decreases the need for high external biases and reduces power consumption.^21,22^ Additionally, incorporating ferroelectric dielectrics improves device sensitivity, allowing for effective modulation of the Schottky barrier height and carrier dynamics, which is particularly beneficial for UV detection. The nonvolatile nature of ferroelectric materials contributes to operational stability, ensuring consistent performance across various conditions, which could benefit the application of memory devices or retention-centric logic.^23–25^ Furthermore, modifying the gate dielectric can help compensate for the low-absorption cross-section of 2D materials, facilitating effective photodetection even at lower light intensities.^26–28^ Finally, these enhancements enable the development of compact device designs that effectively mitigate thermal management issues, broadening the application range of these phototransistors for uses such as environmental monitoring and safety systems.^29–31^

Ultraviolet (UV) photodetectors based on 2D semiconductors often face weak optical absorption and require relatively high operating biases. Ferroelectric dielectrics address both issues: spontaneous polarization supplies a built-in field that aids charge separation and enables nonvolatile electrostatic control of the channel. We therefore develop a polarization-modulated WS_2_/BaTiO_3_ (BTO) phototransistor (back-gated FeFET) in which remanent BTO polarization (∼60 kV cm^–1^) tailors the WS_2_ channel and its Schottky barriers, yielding high responsivity with pA-level dark current at zero external gate bias.

Compared to our earlier work,^32,33^ UV photodetectors were reported based on rGO/BTO, where rGO improved transport across hydrothermally synthesized BTO, and the ferroelectric polarization mainly assisted carrier separation within BTO. Here, we replace rGO with a 2D WS_2_ channel and use the BTO polarization to directly gate WS_2_ (ferroelectric field-effect), producing voltage-free, nonvolatile control of the photoresponse. We also demonstrate robust operation across elevated temperatures and explicitly verify the active ferroelectric phase (tetragonal BTO) via XRD/Rietveld, aligning the structural evidence with device behavior. Our objective is to show that coupling an atomically thin WS_2_ channel to an ultrathin ferroelectric BTO dielectric overcomes the absorption–bias trade-off common to 2D phototransistors by enabling nonvolatile, polarization-programmed photoresponse with strong thermal stability.

We fabricated the barium titanate (BTO) layer using a two-step process: we start with synthesizing BTO nanoparticles through a hydrothermal method, followed by spin-coating these nanoparticles onto a TiO_2_ film grown on titanium foil.^34^ The performance and functionality of the phototransistor were improved by incorporating tungsten disulfide (WS_2_) as the channel.^35,36^ This enhancement resulted in a greater operational efficiency and effectiveness. This study also introduces a novel method for generating bipolar pulses for polarization in an ultrathin BTO ferroelectric film using a back-gated structure. This creates a controlled, nonvolatile, localized electric field (∼60 kV cm^–1^) that effectively modulates the transport properties of the channel and the Schottky barrier. Our findings show that negative capacitance-induced amplification is responsible for the dramatic performance increase and enhanced optical absorption property. This strong internal field efficiently separates photogenerated carriers, effectively compensating for the monolayer WS_2_’s low-absorption cross-section. The result of this low-power operation is evident in the ultralow dark current of 12.3 pA achieved without the need for an external gate voltage, yielding a 20× responsivity increase to 38.5 A/W. This allows for minimal leakage current and low power dissipation. These advancements exceed the performance of standard 2D phototransistors that use ferroelectrics and operate with fixed gate biasing.

## 2 Experimental Section

### 2.1 Synthesis of Barium Titanate Nanoparticles

First, we grew the titanium dioxide (TiO_2_) using 50 mL of titanium­(III) chloride (TiCl_3_) with 12 mL of hydrochloric acid (HCl), and then this mixture, named Solution A, was added to an autoclave at 12 kg/cm^2^ at 180 °C (Figure 1). Then, the grown TiO_2_ solution was dried at 80 °C to prepare TiO_2_ nanoparticles (NPs). The TiO_2_ NPs are mixed with another solution named Solution B, containing 42.56 g of Ba (OH)_2_. 8H_2_O crystals were dissolved in 160 mL of DI water. The above mixture is then added to the autoclave of having 27 kg/cm^2^ at 225 ± 2 °C. Then, the grown BTO solution is dried at 100 °C to prepare the BTO NPs.

**Figure 1:**
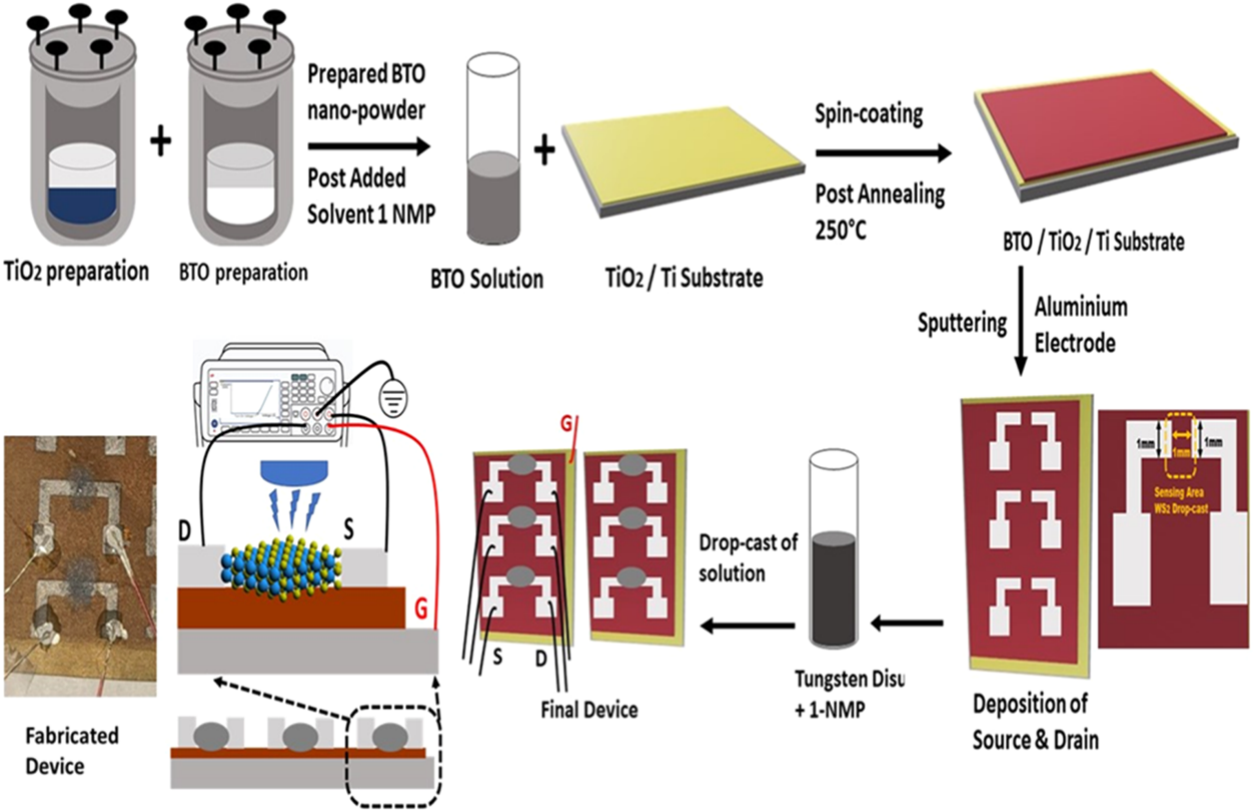
Fabrication of the studied FeFET.

### 2.2 Fabrication of Phototransistors

TiO_2_ film was synthesized hydrothermally on a titanium sheet, which acted as the substrate. The hydrothermal growth of the TiO_2_ film was identical when using the aforementioned Solution A. Following this, barium titanate nanoparticles (BTO NPs) were prepared and then spin-coated onto the TiO_2_ substrate using 1-*N*-methyl-2-pyrrolidone (1-NMP) as the solvent, maintaining a concentration ratio of 12.5 mg/mL. To ensure proper mixing, the prepared solution was ultrasonicated for 2 h. The spin-coating process was carried out at 1500 rpm for 5 min, after which the coated substrate was dried in an oven at 80 °C. With the TiO_2_-based substrate featuring BTO complete, two aluminum electrodes were deposited as the phototransistor’s source and drain onto the BTO/TiO_2_ structure using sputtering for 15 min at a DC power setting of 100 W.

### 2.3 Fabrication of 2D WS_2_ Channel in Phototransistors

We fabricated a phototransistor with the structure Al/BTO/TiO_2_ and subsequently modified it by incorporating a two-dimensional material called tungsten disulfide (WS_2_), which was purchased from Sigma-Aldrich (catalog no. 243639) as a 2 μm, 99% pure powder. To prepare the WS_2_, we added it to the solvent 1-*N*-methyl-2-pyrrolidone (1-NMP) at a concentration of 1.5 mg/mL, then thoroughly mixed the solution through ultrasonication for 2 h. Afterward, we centrifuged the mixture at 6000 rpm for 30 min to separate the liquid from any unwanted residues. The resulting solution was then applied using the drop-casting technique, where 0.5 μL was deposited between the two aluminum electrodes (source and drain), having an area approximately of 1 mm × 1 mm in the Al/BTO/TiO_2_ structure as shown in Figure 1. At last, the phototransistor with WS_2_ was dried on a hot plate at 70 °C.

## 3 Results and Discussion

### 3.1 Material Characterization

Figure 2 includes the BTO/TiO_2_/Ti pattern (Figure 2a) alongside WS_2_/TiO_2_/Ti (Figure 2b) and WS_2_/BTO/TiO_2_/Ti (Figure 2c), with all peaks indexed. The BTO film matches tetragonal BaTiO_3_ (*P*4*mm*), with reflections at ∼22.3° (100), 31.3° (110), 38.9° (111), 45.3° (200), 50.4° (210), 56.1° (211), and 65.9° (220), confirming the ferroelectric phase used in the device.

**Figure 2:**
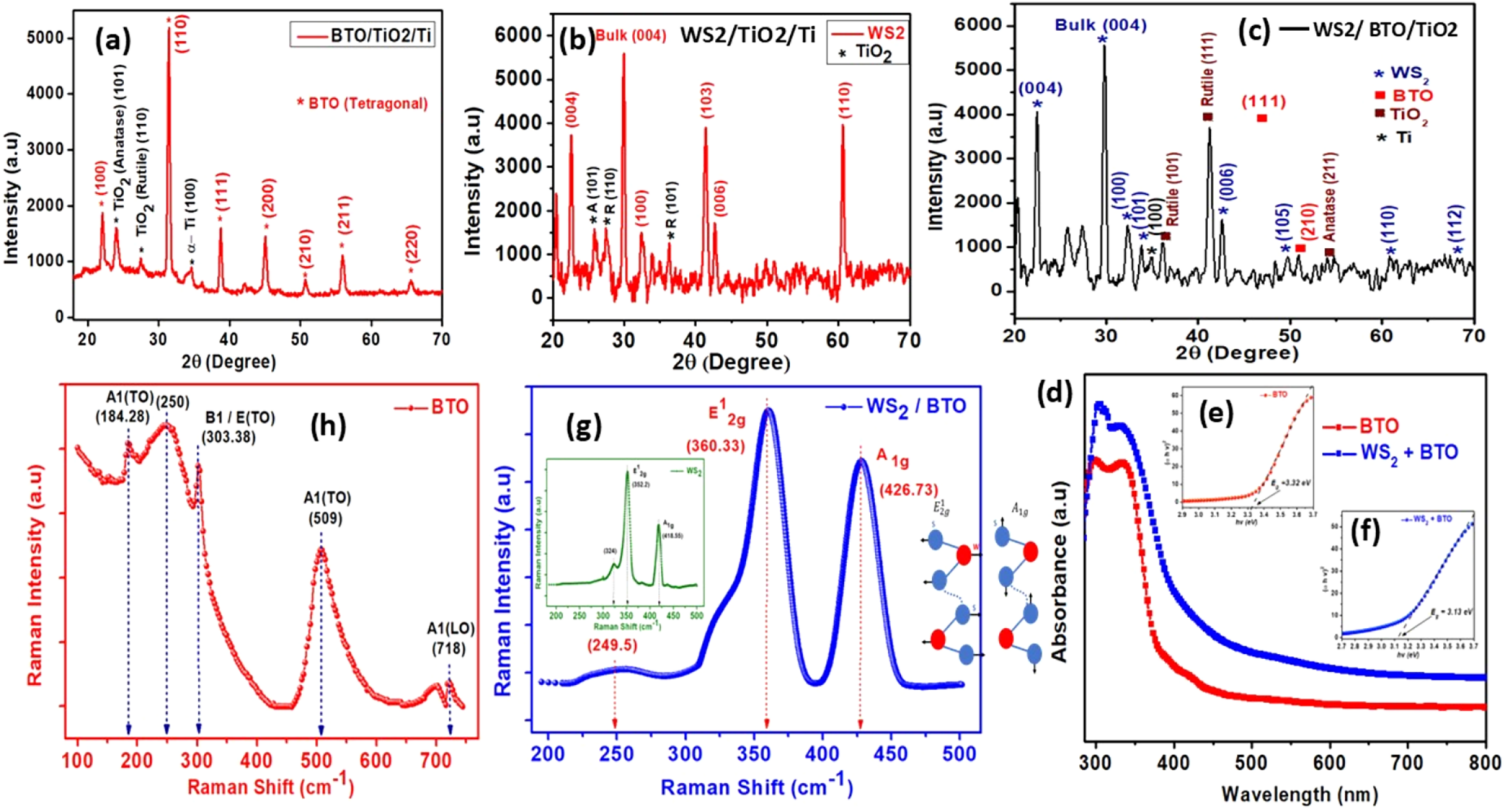
XRD spectrum of (a) BTO, (b) WS_2_, (c) WS_2_/BTO; UV–vis spectroscopy (d) absorbance pattern and Tauc Plot of (e) BTO, (f) WS_2_/BTO; and Raman Shift of (g) BTO (h) WS_2_/BTO.

Full-pattern Rietveld refinements (10–70° 2θ, Cu Kα) confirm that both the hydrothermally grown BTO film and the WS_2_/BTO heterostructure contain the tetragonal BaTiO_3_ (*P*4*mm*) phase. The patterns were modeled by t-BTO + 2H-WS_2_ + TiO_2_ (anatase ± rutile), reproducing all of the observed peaks without residuals. The refined lattice parameters (*a* = 4.0045 Å, *c* = 4.2006 Å, *c*/*a* = 1.049) verify the ferroelectric tetragonal distortion. Refinement statistics (*R*_wp_ = 50.1%, *R*_p_ = 36.4%, and χ^2^ = 0.0157 for BTO; *R*_wp_ = 48.5%, *R*_p_ = 37.6%, and χ^2^ = 0.0393 for WS_2_/BTO) are typical for thin polycrystalline films on Ti substrates. The low χ^2^ and flat residuals indicate that the derived lattice parameters are reliable and that WS_2_ integration does not alter the ferroelectric structure.

The UV–vis absorbance spectra demonstrate the optical behavior of BTO and the WS_2_–BTO composite in response to different light wavelengths. For pure BTO, the spectrum shows that it absorbs strongly in the UV region, indicative of its wide bandgap (∼3.32 eV). This supports BTO’s function as a photocatalyst under UV light, while also highlighting its limitations in utilizing visible light, as it cannot absorb photons with lower energy. The WS_2_–BTO composite (Figure 2d) shows enhanced absorption in the visible region, suggesting that incorporating WS_2_ improves the material’s ability to utilize visible light. This improvement arises because WS_2_, a transition metal dichalcogenide, has a narrower bandgap (∼1.35 eV), enabling it to absorb photons in the visible and near-infrared ranges. The synergistic interaction between WS_2_ and BTO likely generates a composite with a reduced overall optical bandgap (∼3.13 eV), broadening its optical response.

The Tauc plot is a graphical method to estimate the bandgap energy of optical materials. Figure 2e indicates that barium titanate oxide (BTO) has an extrapolated bandgap of 3.32 eV, classifying it as a wide bandgap semiconductor. This property results in strong intrinsic absorption in the ultraviolet (UV) range (λ ≤ 373 nm), making BTO an ideal candidate for high-performance UV photodetectors and phototransistors. The WS_2_–BTO composite (Figure 2f), however, shows a reduced bandgap (∼3.13 eV), demonstrating that the incorporation of WS_2_ results in bandgap narrowing. The composite develops a reduced effective optical bandgap due to the formation of a Type-II (Staggered) heterojunction at the WS_2_/BTO interface. Pure BTO is a wide bandgap semiconductor (∼3.32 eV), absorbing only UV light. Integrating the narrower bandgap, WS_2_ (∼1.35 eV) aligns its conduction and valence band edges in a staggered configuration relative to BTO. This band arrangement is crucial as it creates a path for interfacial charge transfer (ICT) transitions. Specifically, ICT allows a lower-energy visible photon to excite an electron from the WS_2_ valence band (WS_2_-VB) to the BTO conduction band (BTO-CB). This cross-gap transition dictates a new, smaller effective optical bandgap for the entire heterostructure, significantly widening the composite’s optical absorption spectrum into the visible region. Critically, this spatial charge separation into different materials effectively extends carrier lifetimes by suppressing rapid electron–hole recombination, enhancing the device’s overall photosensitivity.

Raman spectroscopy reveals information about lattice vibrations and material phase.^37^ For BTO, peaks such as A1­(TO), B1/E­(TO), and A1­(LO) correspond to vibrations in the tetragonal phase, as shown in Figure 2h. These modes arise from the symmetric and asymmetric vibrations of TiO_6_ octahedra in the BTO lattice. The observed peaks confirm the structural integrity and retention of BTO’s polar tetragonal phase, which is essential for the material’s ferroelectric and photocatalytic properties. The broad range of Raman-active phonon modes directly reveals the material’s complex lattice dynamics. These intricate atomic vibrations represent fundamental lattice characteristics that fundamentally explain the markedly high dielectric permittivity and robust polarization properties. The unique properties of this material make it a great candidate for advanced electronic and photonic applications. This includes high-performance ferroelectric devices, new sensing platforms, and energy-efficient memory technologies.

Turning to Figure 2g, the Raman spectrum of the WS_2_–BTO composite is displayed, emphasizing the presence of WS_2_ within the composite material. The distinct peaks at 360.33 cm^–1^ (E_2g_^1^) and 426.73 cm^–1^ (A_1g_) are characteristic of WS_2_ vibrational modes. The E_2_^1^_g_ peak corresponds to the in-plane vibrational mode, where tungsten (W) and sulfur (S) atoms vibrate in the same direction, while the A_1_g peak is associated with the out-of-plane vibrational mode, where sulfur atoms vibrate in opposite directions. The inset in Figure 2(f) provides a closer view of the WS_2_ Raman peaks, affirming its structural integrity even after being integrated with BTO. Furthermore, an additional peak is seen at 249.5 cm^–1^. The observation of these Raman-active modes in the composite suggests that WS_2_ enhances light absorption and charge carrier dynamics within the material system, potentially improving its performance in optoelectronic applications. The coexistence of both BTO and WS_2_ peaks in the composite spectrum confirms that the materials retain their structural characteristics, which are essential for achieving the desired functional properties.

The ability of the WS_2_–BTO composite to function effectively across UV and visible light regimes can be explained by the concept of charge separation at a heterojunction interface. When light is absorbed by the composite, photogenerated electrons and holes are created. In this system, electrons from WS_2_ are likely transferred to BTO due to the alignment of conduction band edges between the two materials, while holes remain in WS_2_. This separation reduces the recombination rate of charge carriers, increasing the lifetime of photogenerated carriers and improving the material’s photocatalytic performance. Moreover, the heterostructure formed by WS_2_ and BTO enhances the built-in electric field at their interface.

### 3.2 Ferroelectric Response of WS_2_/BTO Heterostructures under Cycled Bipolar Triangular Poling

To evaluate the ferroelectric response, two fabricated phototransistors were tested: one incorporating only a BTO layer and the other based on a WS_2_/BTO heterostructure. The FIB cross-sectional view of the WS_2_-integrated phototransistor, showing the layered structure from the top aluminum electrodes down to the bottom titanium foil substrate, is presented in Figure 3a. A cyclic bipolar triangular voltage signal (50 Hz, 50 cycles) was applied between the drain and source aluminum electrodes of each device (Figure 3b). This poling configuration enabled repeated polarization switching, allowing the extraction of polarization–electric field (P–E) hysteresis loops for both structures and providing insights into the influence of WS_2_ integration on ferroelectric switching behavior.

**Figure 3:**
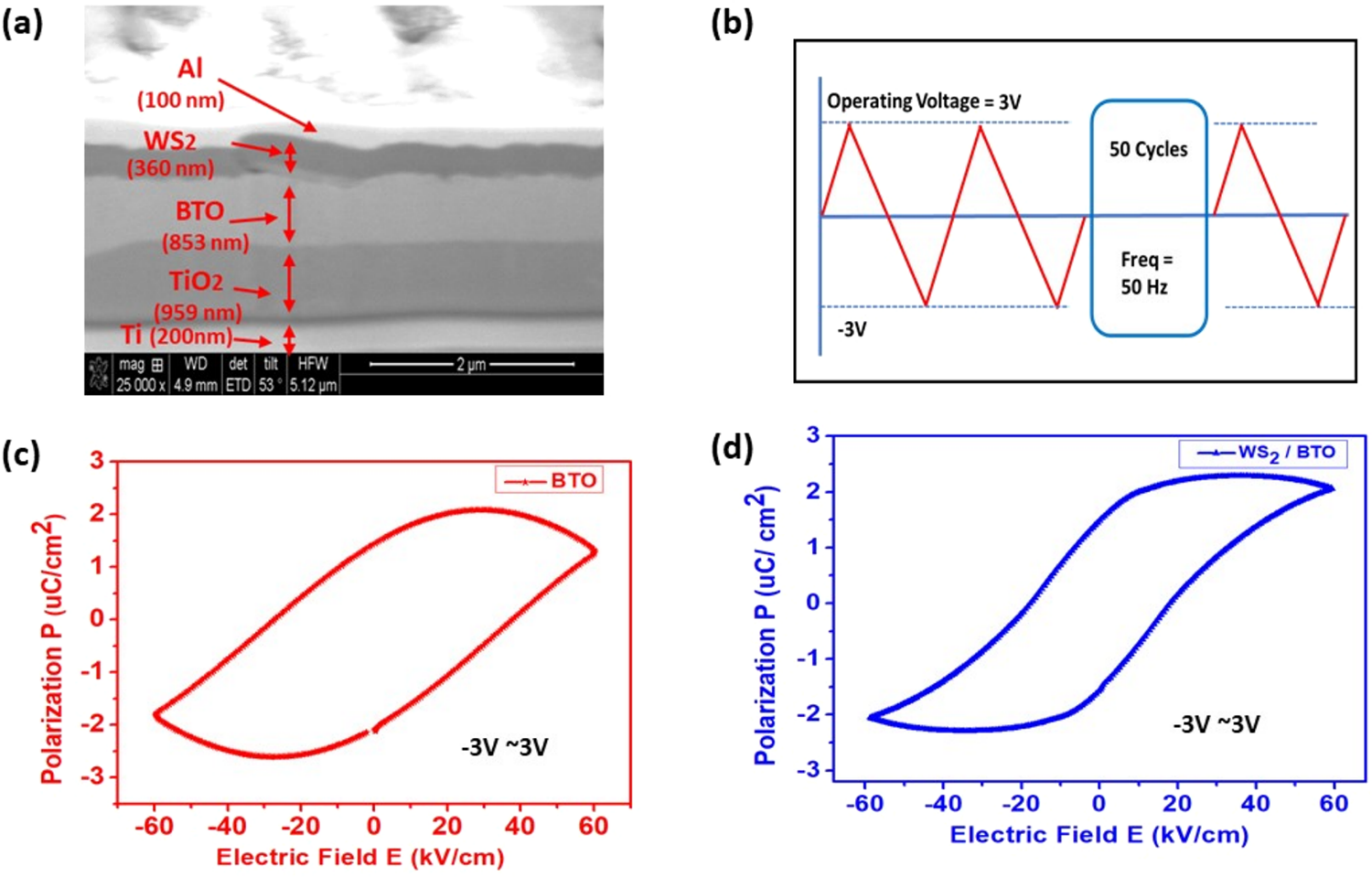
(a) Cross-sectional view of the fabricated FeFET structure. (b) Applied cyclic poling voltage to the device. (c) Resulting PE curve of the bare BTO-structured FeFET. (d) Resulting PE curve of the FeFET with BTO/WS_2_ structure.

In the metal-ferroelectric-semiconductor (MFS) structure using bare BTO, the measured coercive field is 40 kV/cm and the remanent polarization is 2.5 μC/cm^2^ (Figure 3c). These values indicate that BTO retains a strong ferroelectric response, requiring a relatively high electric field to switch polarization. After the incorporation of WS_2_ into the BTO structure, both Ec and Pr decrease, with Ec reducing to 20 kV/cm and Pr decreasing to 2.0 μC/cm^2^ (Figure 3d). The introduction of WS_2_ into the bare BTO structure significantly affects its ferroelectric properties, specifically the coercive field (Ec) and remanent polarization (Pr). This shift suggests that WS_2_ modifies polarization switching behavior, likely through enhanced interfacial screening or charge redistribution that influences domain wall dynamics and switching kinetics. The reduced Ec indicates that a lower voltage is sufficient for polarization reversal, while the diminished Pr reflects a decrease in the ferroelectric charge storage capacity. These changes are attributed to the interfacial role of WS_2_, which alters the local electric field environment, improves charge compensation, and perturbs dipole alignment within the BTO layer. Collectively, these interfacial effects facilitate more efficient polarization switching and enhance the overall tunability, presenting a viable pathway toward reduced power consumption and improved energy efficiency in advanced ferroelectric memory and sensing applications.

### 3.3 Drain–Source *I*–*V* Characteristics Modulated by Ferroelectric Poling in BTO and WS_2_/BTO Phototransistors

To evaluate how different ferroelectric polarization states influence charge transport behavior, we investigated the *I*–*V* characteristics of BTO and WS_2_/BTO FET photodetectors under three distinct poling conditions: positive DC bias (up poling), negative DC bias (down poling), and bipolar triangular cyclic poling (50 Hz, 50 cycles). These measurements were conducted to evaluate the polarization-dependent electrical response and to investigate how ferroelectric polarization modulates the charge transport between the drain and source electrodes. The triangular cyclic poling condition enables the observation of full polarization switching behavior, while the static DC poling conditions highlight the effects of aligned remanent polarization states on carrier transport. Together, these measurements provide insights into how field-induced polarization states influence device sensitivity and current flow, supporting the optimization of the device architecture and biasing schemes for polarization-tunable optoelectronic applications.

The current vs voltage measurements were conducted using the Keithley B1500A semiconductor analyzer within a probe station equipped with a vacuum chamber, ensuring a stable light environment where the sample was positioned on the platform inside the chamber. The observed measurements demonstrate the impact of different poling conditions on the electrical properties of BTO and WS_2_/BTO samples. Testing under various states—Cyclic Polled, Upward Polled (+10 V DC bias for 30 s), and Downward Polled (−10 V DC bias for 30 s) allowed for a comprehensive evaluation of the *I*–*V* characteristics across a drain-to-source voltage range of −1 to +1 V. The Cyclic polled condition indicates the state. This thorough evaluation is crucial for tailoring the devices’ electrical properties, enhancing responsivity, and minimizing dark current by leveraging the effects of polarization on charge transport mechanisms.

As highlighted in Figure 4a, the polarization states of BTO illustrate the transition from a fresh state, characterized by random dipole orientations, to an Up Poled State, where dipoles align upward, thereby enhancing conductivity. Conversely, in the Down Poled State, dipoles align downward, resulting in reduced conductivity. The semilogarithmic graph in Figure 4b illustrates that, for the BTO sample under various poling conditions, the open-circuit voltage varied from 0 to −0.3 V, while the short-circuit current exhibited a range from 10 nA to 50 pA. In comparison, the WS_2_/BTO sample presented an open-circuit voltage of 0.1 to 0.05 V and a short-circuit current from 2 μA to 5 pA as depicted in Figure 4c.

**Figure 4:**
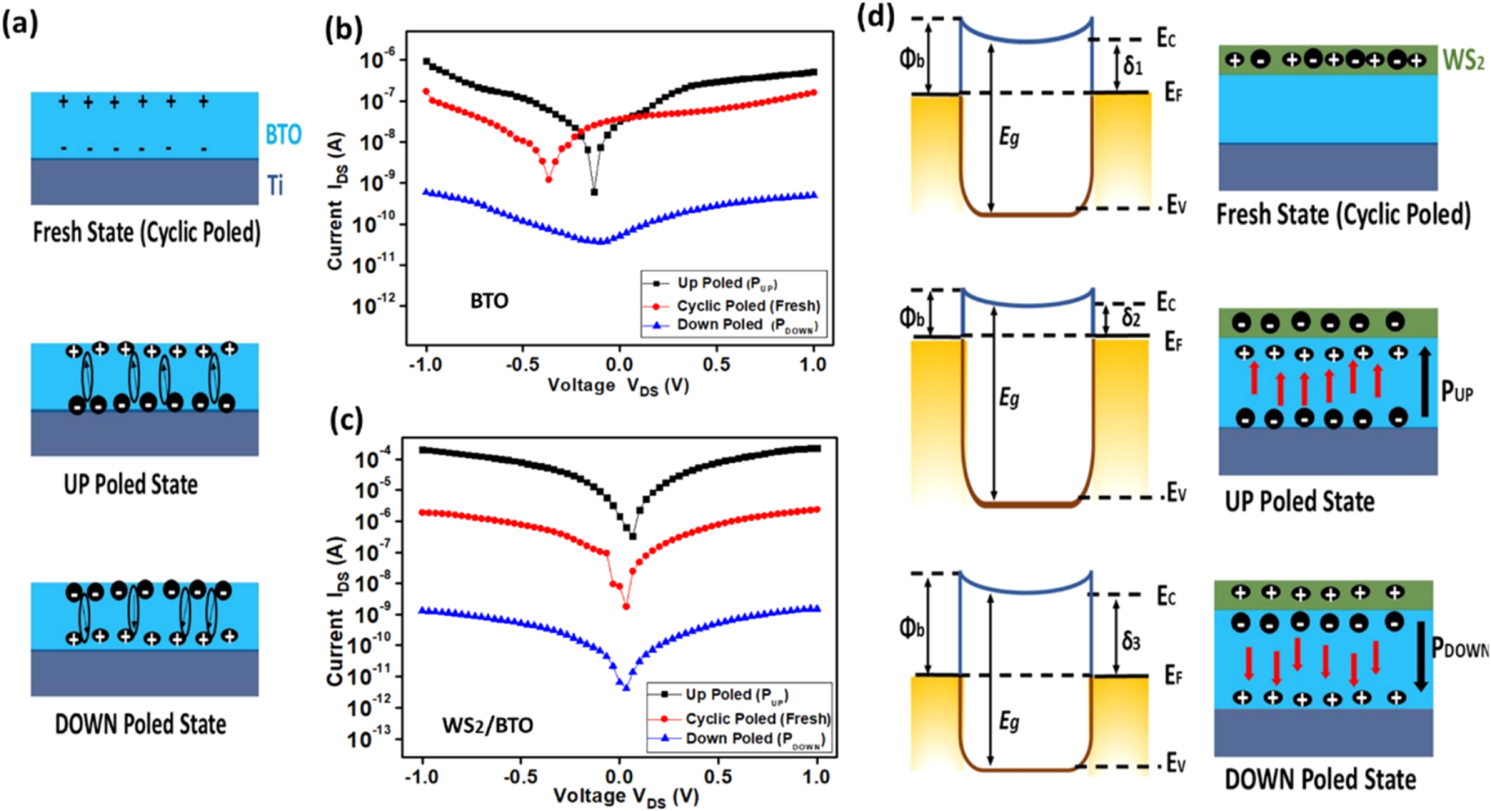
(a) Illustration of the investigated poled states. (b) IV curve of the bare BTO-structured FeFET for different poled states. (c) IV curve of the BTO/WS_2_ structured FeFET for different poled states. (d) Band diagrams elucidate different polarized states of the FeFET.

In the Fresh State (Cyclic Poled), the band diagram of WS_2_ demonstrates a stable charge distribution with well-defined conduction (Ec) and valence (Ev) bands, which are critical for electronic transport (Figure 4d). The Up Poled state causes a shift in the band diagram, resulting in positive charge accumulation near the top metal contact, which significantly enhances the hole density and conductivity. On the other hand, the Down Poled state modifies the band diagram further by shifting positive charges toward the bottom contact, altering the Fermi level and creating a unique charge environment that may reduce conductivity compared to the Up state but offers distinct advantages for specific electronic applications.

Overall, these insights into band diagrams and *I*–*V* characteristics under varying poling conditions elucidate how polarization affects charge transport mechanisms, providing a foundation for optimizing the electrical performance of the BTO and WS_2_/BTO devices.

### 3.4 Ferroelectric Polarization-Induced Modulation of BTO and WS_2_/BTO Phototransistor Characteristics

In conjunction with these findings, the transfer characteristics *I*_DS_ vs *V*_GS_ of a WS_2_-based field-effect transistor (FET) featuring a BTO dielectric layer are depicted in Figure 5a, with a source–drain voltage *V*_DS_ maintained at 0.5 V, while the gate voltage *V*_GS_ varies from −3 to 3 V. The observed characteristics reveal a clockwise trend indicative of typical n-type behavior, with the drain current IDS measured at approximately 10^–6^ A for bare BTO and around 10^–5^ A for the WS_2_/BTO structure, indicating a significant increase in current due to the integration of the 2D material (Figure 5d).

**Figure 5:**
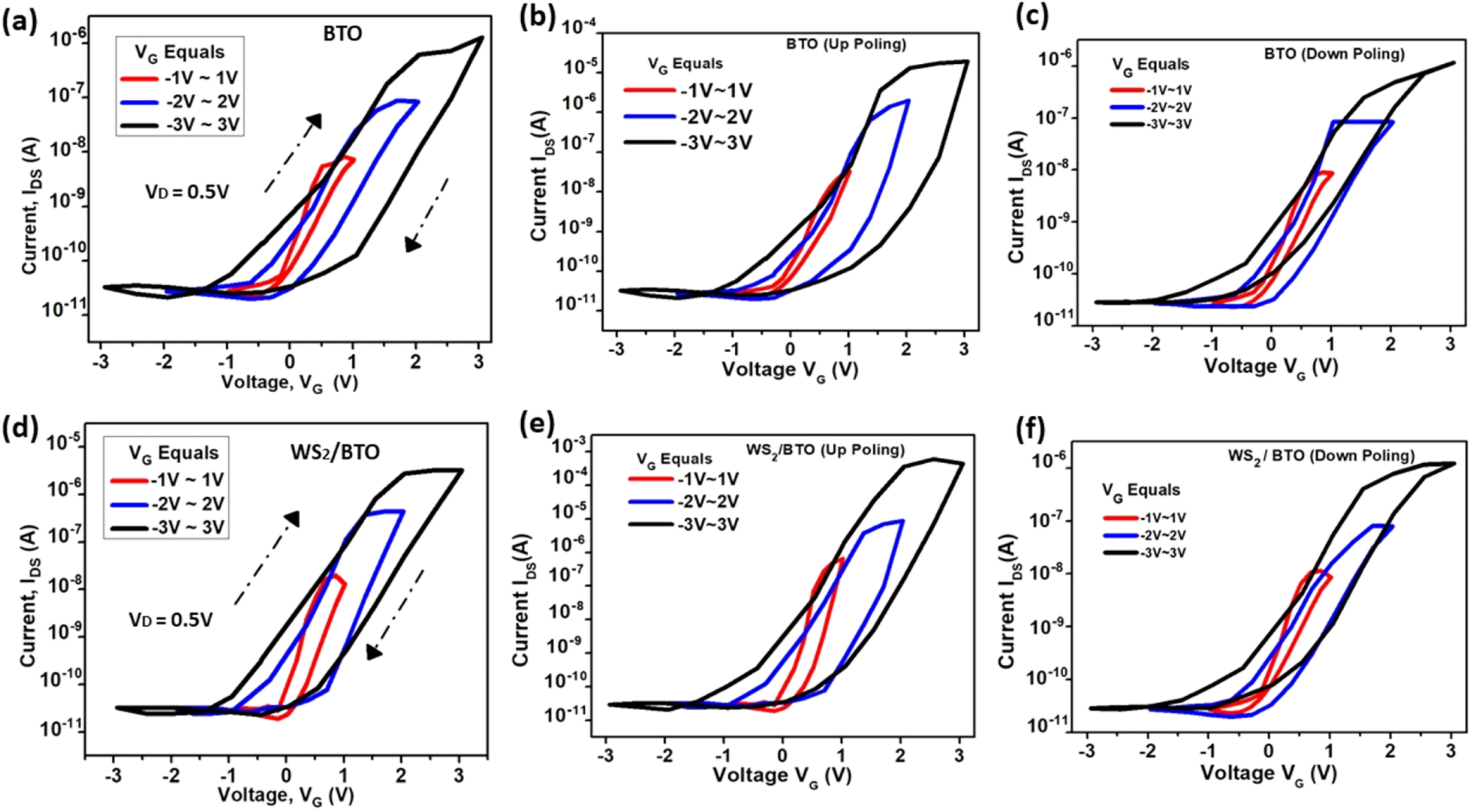
Gate modulation in different voltage ranges from −3 to 3 V at different poling conditions for (a–c) the BTO device and (d–f) the WS_2_/BTO device.

This increase in drain current signifies enhanced carrier mobility and improved gate control, attributable to the ultrathin nature of 2D materials, which allows for a stronger electrostatic influence. Consequently, this results in higher current levels at reduced gate voltages, potentially leading to a lower threshold voltage and an improved on/off current ratio, which together contribute to superior device performance.

Furthermore, the clockwise hysteresis loop appears, primarily due to dynamic charge trapping and detrapping occurring in the defect states within the material. Charges can become temporarily trapped at defect sites in the dielectric or semiconductor layers, creating a lag in response during the voltage sweep. As the gate voltage is varied, these trapped charges alter the effective gate voltage experienced by the channel, leading to shifting current levels that produce the hysteresis effect. Such charge dynamics are critical for understanding the device’s stability, reliability, and operational performance.

Additionally, Figure 5b,c illustrates the transfer characteristics of the bare BTO device under up poling and down-poling conditions, respectively. In the up poling state (Figure 5b), a positive polarization is induced in the BTO layer prior to measurement, resulting in a rightward shift in the hysteresis window and an enhanced electron accumulation with a current value range of 10^–5^ A (approximately 30 μA). Conversely, the down-poling state (Figure 5c) introduces a negative polarization, leading to a leftward shift, indicating suppressed electron accumulation with a current value in the range of 10^–6^ A (approximately 1.2 μA). Similar trends are observed in the WS_2_/BTO heterostructure, as shown in Figure 5e,f. Under up poling conditions (Figure 5e), the device demonstrates an increased current of 10^–3^ A (1 mA) due to improved gate–channel coupling facilitated by the ferroelectric polarization. In contrast, down-poling (Figure 5f) is consistent with reduced gate-induced charge accumulation, resulting in a decrease in the current to 10^–6^A (approx 1.41 μA). These results underscore the critical role of ferroelectric polarization in modulating the electronic characteristics of the device.

Similar behaviors have been reported in FETs incorporating two-dimensional materials such as MoS_2_ and graphene, particularly when combined with back-gated ferroelectric layers, underscoring persistent challenges in interface stability and polarization control at the nanoscale. The WS_2_/BTO heterostructure offers key advantages over BTO alone, including a reduced coercive voltage for polarization switching, which enhances energy efficiency and facilitates integration into low-power systems. Additionally, WS_2_ can improve the dielectric response and carrier mobility, supporting the development of flexible, lightweight electronics with enhanced sensing performance. Notably, WS_2_ may also stabilize the ferroelectric behavior at elevated temperatures. This effect is attributed to its intrinsic thermal robustness and efficient interfacial charge screening, which suppresses depolarization fields and minimizes internal electric field fluctuations. As a result, dipole alignment is better maintained under thermal stress, extending the operational temperature range and improving device reliability.

### 3.5 Photodetection Measurement Setup and Photocurrent Response of Devices under UV Irradiation with Varying Poling Conditions

#### 3.5.1 Photodetection Measurement Setup

The measurement setup for a phototransistor consists of several crucial components that collectively facilitate the accurate measurement of the device’s response, particularly to UV light (Figure 6). At its core, an isolated chamber houses the phototransistor, providing a controlled environment that minimizes external light interference and allows for the precise regulation of atmospheric conditions such as temperature and humidity. A power source supplies the necessary voltage, specifically setting the drain voltage (*V*_ds_) to 0.5 V, which is essential for biasing the phototransistor appropriately during measurements.

**Figure 6:**
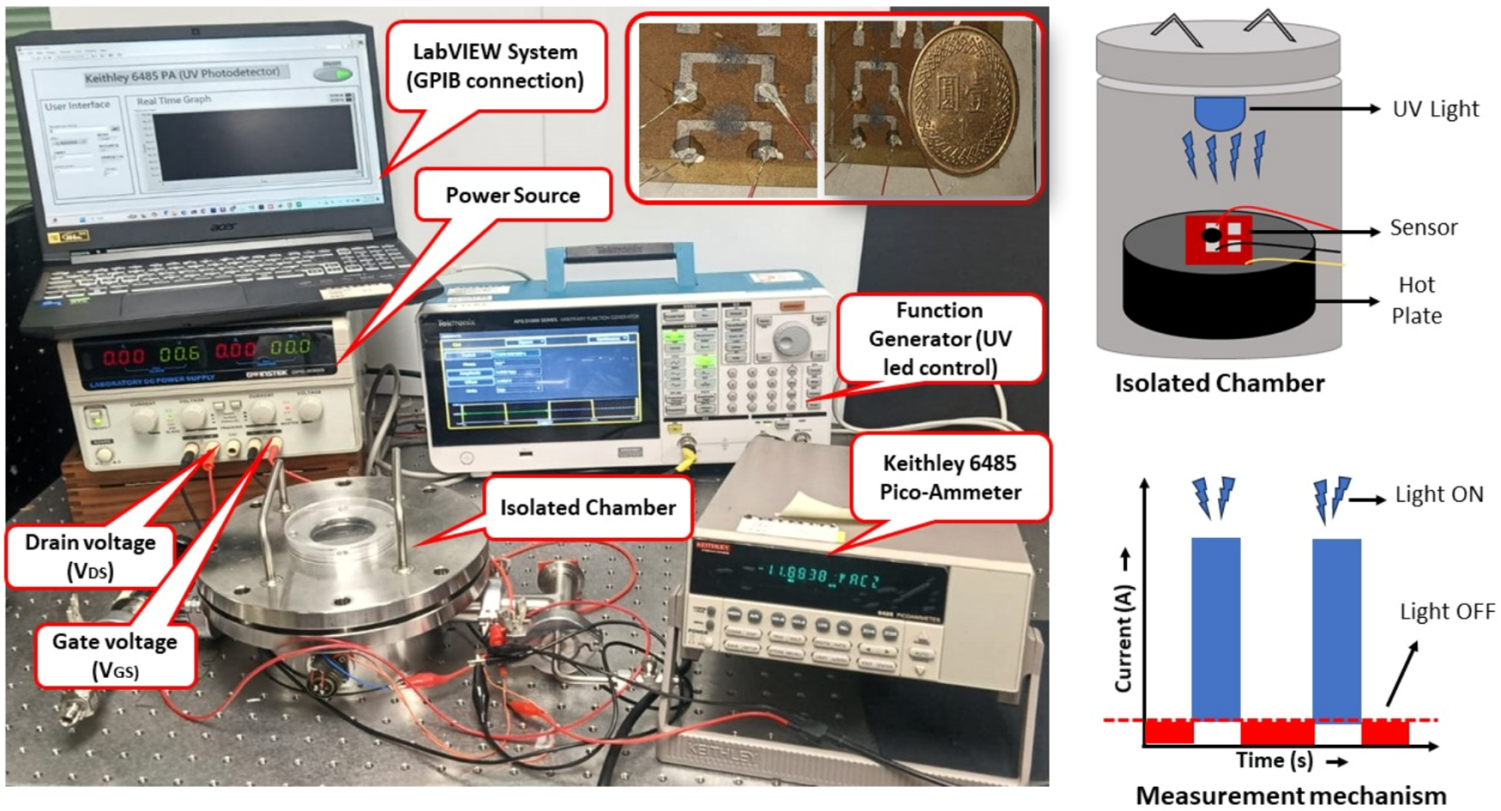
Measurement setup of FeFET for UV photodetectors with different voltage values of *V*_DS_ and *V*_GS_.

Central to the operation is a function generator, which generates the control signal for the UV light source. This device modulates the ON and OFF states of the UV LEDs illuminating the phototransistor for 3.5 s each; the square signal is applied to the UV light having a 7 s period (3.5 s ON and 3.5 s OFF). The output current produced by the phototransistor is measured using a Keithley 6485 Pico-Ammeter, which is designed for high sensitivity, ensuring that even small currents can be detected reliably. Additionally, a LabVIEW system is integrated into the setup, allowing for real-time data acquisition and analysis through a GPIB connection, which facilitates the visualization of measurement results as the experiments progress.

The measurement procedure begins with setup calibration, ensuring all connections are intact and initializing the system by setting the gate voltage (*V*_gs_) to 0 V while confirming that the drain voltage (*V*_ds_) remains at 0.5 V. Once operational, the function generator activates the UV light—commonly in a square wave pattern, enabling the phototransistor to respond to light intensity changes. Concurrently, the pico-ammeter records the generated current, capturing peaks that correlate with the UV light’s ON state and subsequent drops when the light is OFF.

The measurement mechanism demonstrates a clear relationship between the UV light being ON (resulting in an increase in current) and OFF (leading to a decrease). This setup and methodical analysis provide a comprehensive framework for evaluating the phototransistor’s efficiency and sensitivity under controlled lighting conditions, thereby offering valuable insights into its operational characteristics.

#### 3.5.2 Photocurrent Responses and Responsivity of the Phototransistor under UV Light with Varying DC Poling Conditions

The integration of WS_2_ with BTO and subsequent polarization demonstrate remarkable improvements in photodetector performance. In bare BTO phototransistors, cyclic polarization increases the photocurrent from 20 nA to 0.8 μA under the *V*_GS_ = 0 condition, while the WS_2_/BTO heterostructure shows a dramatic enhancement from 0.1 to 22.5 μA after polarization. This significant 28-fold increase in photocurrent (from 0.8 to 22.5 μA) by adding WS_2_ to BTO can be attributed to improved charge carrier dynamics and enhanced ferroelectric polarization effects (Figure 7a).

**Figure 7:**
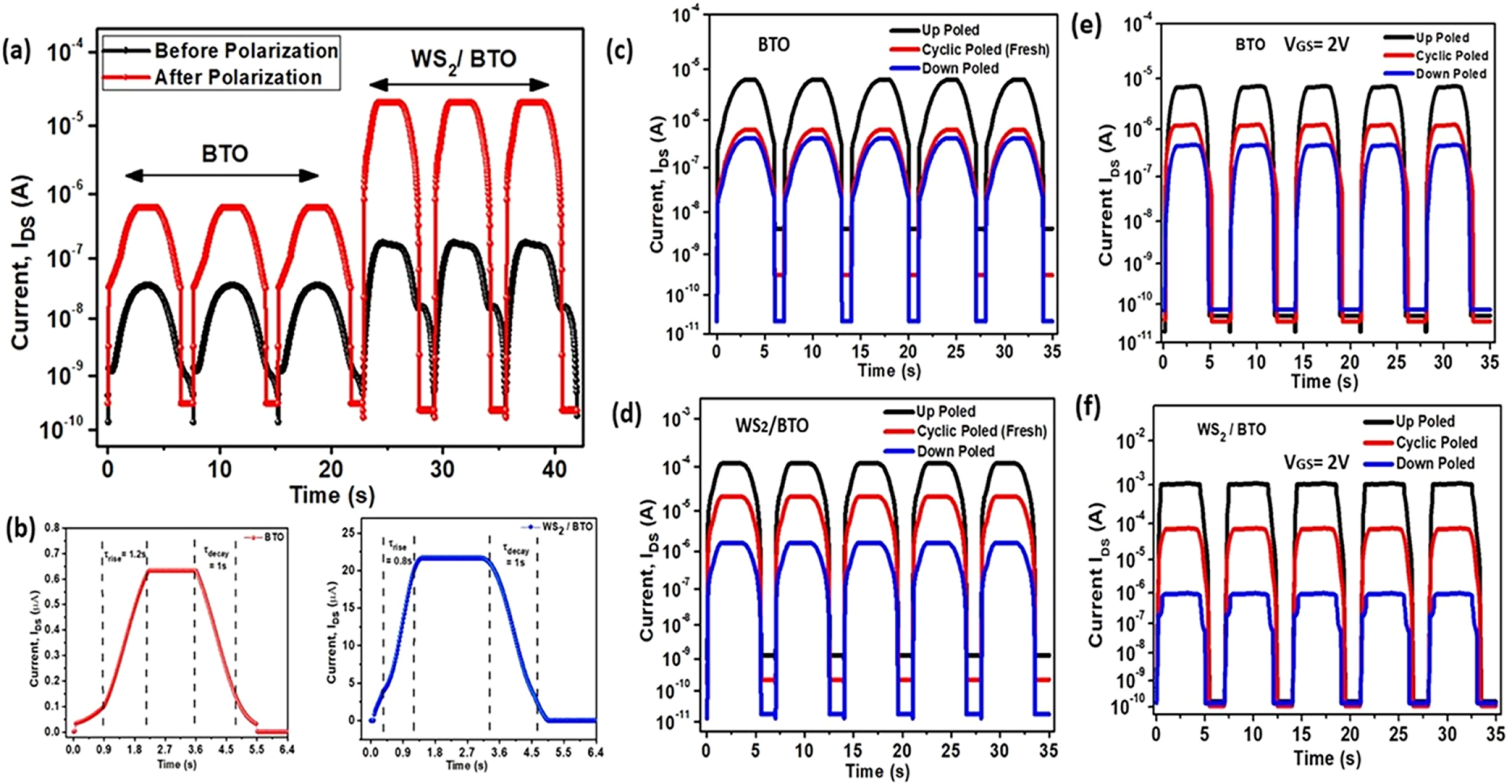
Current response (*I*–*T*) *I*_DS_ of (a) BTO and WS_2_/BTO before–after polarization, (b) response time, (c) DC poling effect of the BTO device, and (d) DC poling effect of the WS_2_/BTO device. Current response (*I*–*T*) *I*_DS_ at different poling conditions with fixed voltage to *V*_GS_ = 2 V (e) BTO, (f) WS_2_/BTO.

The responsivity, *R*, is the crucial factor to be measured for a photodetector. The equation used for evaluating the responsivity is

$$R=\frac{I_{\mathrm{photo}-I_{\mathrm{dark}}}}{A\times P_{\mathrm{inc}}}$$

where *I*_Photo_ is the photocurrent, *I*_dark_ is the dark current, *A* is the area of the sensing area, and *P*_inc_ is the power of the incident light. The power of the UV light source was 6 μW/cm^2^ (375 nm), with the sensing area being 10 mm^2^. The observed responsivity of the sensor was 1.87 (∼1.9) A/W for bare BTO, but for WS_2_/BTO, the responsivity was 38.49 A/W. The responsivity condition was tested after the cyclic poled conditions of both devices.

The poling-dependent analysis reveals even more striking differences. In bare BTO, the upward poled state generates a photocurrent of 7 μA with a dark current of 7 nA, while cyclic poling produces 0.7 μA photocurrent (0.8 nA dark current) and downward poling yields 0.5 μA photocurrent (70 pA dark current) (Figure 7c). The WS_2_/BTO heterostructure demonstrates superior performance with upward poling generating 0.1 mA photocurrent (2 nA dark current), cyclic poling producing 12 μA photocurrent (0.5 nA dark current), and downward poling yielding 1 μA photocurrent (10 pA dark current) (Figure 7d). Additionally, the device shows improved response times, with τ_rise_ decreasing from 1.2 s to 0.8 s after WS_2_ addition, resulting in a 50% reduction in the τ_rise_/τ_decay_ ratio (from 1.2 to 0.8) (Figure 7b).

In contrast to the zero gate-bias condition (Figure 7c,d), a significant enhancement in current levels (*I*_DS_) is observed upon applying a gate voltage of *V*_GS_ = 2 V (Figure 7e,f). Specifically, the photocurrent in the bare BTO device increases from 7 to 9 μA under upward poling (Figure 7e), while the WS_2_/BTO heterostructure exhibits a much more pronounced increase, from 0.1 to 1.05 mA (Figure 7f). This substantial improvement highlights the strong synergistic effect of WS_2_ and gate-induced electrostatic modulation. Moreover, after application of *V*_GS_ = 2 V, the dark current becomes more stable and exhibits reduced variation between polarization states, indicating effective suppression of polarization-induced fluctuations. This stabilization is attributed to enhanced carrier screening and a more uniform channel potential under gate bias. Furthermore, a clearer distinction between up- and down poled states is achieved, particularly in the WS_2_/BTO device, confirming that *V*_GS_ not only amplifies the photocurrent but also significantly improves polarization-dependent current modulation, which is crucial for reliable ferroelectric and optoelectronic device operation.

The dark current is fundamentally determined by both the BTO’s polarization state and the device structure. In the BTO-only device (Figure 7c,e), the polarization field directly dictates the current: the “Down Poled” state maximizes the interfacial barrier height, thus suppressing leakage current, while the “Up Poled” state minimizes it. However, the most significant dark current reduction comes from incorporating the WS_2_ layer (Figure 7d,f). The ferroelectric field, working across the WS_2_/BTO interface, induces strong carrier depletion in the WS_2_ channel, effectively creating a high Schottky barrier that prevents current flow. This mechanism maintains an extremely low dark current, critical for a high signal-to-noise ratio. Crucially, the WS_2_/BTO device sustains this low leakage even under an applied gate bias—a condition that typically enhances carrier injection, confirming the powerful suppression of the dark current enabled by the synergistic effect of the ferroelectric gate and the WS_2_ channel.

These enhancements stem from multiple synergistic mechanisms, particularly when combining 2D materials with cyclic poling. The process creates improved domain alignment and stronger interfacial coupling between layers, leading to enhanced polarization control and a better charge distribution. The 2D material serves as an effective charge modulation layer, while cyclic poling establishes uniform domain patterns and reduces switching voltage requirements. This powerful combination results in superior retention characteristics, reduced fatigue, and enhanced electromechanical responses. The stable ferroelectric properties and optimized charge transport mechanisms make these devices particularly promising for advanced sensing and memory applications, demonstrating the significant potential of integrating 2D materials with ferroelectric substrates for next-generation electronic devices.

### 3.6 Photocurrent Response of Devices under Various Temperatures with Poling Effect

BTO (barium titanate) is a special material that responds to both light and temperature changes, making its behavior as a UV photodetector temperature-dependent. At room temperature (when d*T*/d*t* = 0), the electric dipoles maintain relatively stable positions with only minor random movements, keeping the overall polarization constant in a state of equilibrium. However, when the temperature increases (d*T*/d*t* > 0), these dipoles become more energetic and begin moving more vigorously, gradually losing their aligned positions. This increased thermal activity leads to three significant effects: reduced overall polarization in the material, decreased charge collection on the BTO surface, and creation of an electrical voltage across the material. This behavior is analogous to an organized crowd (representing aligned dipoles at room temperature) becoming increasingly disorganized as the conditions change (representing higher temperatures). The temperature sensitivity of BTO directly impacts the device’s UV detection performance, making it a crucial consideration for practical applications where temperature variations may occur.

In a transistor-based UV photodetector, this effect becomes even more significant, as BTO is often used as a dielectric or active layer, influencing the channel conductance of the field-effect transistor (FET). Under UV illumination, charge carriers are generated, and their transport is modulated by the dipole alignment in the BTO layer, but as the temperature increases, the reduction in polarization weakens this modulation effect, decreasing photoresponse efficiency. Additionally, dipole disorder in BTO can shift the transistor’s threshold voltage, requiring different gate biases to maintain optimal performance. Furthermore, charge trapping at the BTO–semiconductor interface can introduce more scattering and reduce carrier mobility, which degrades the overall transistor response. Since phototransistors rely on charge trapping and recombination dynamics for signal amplification, temperature fluctuations in BTO can alter carrier lifetimes, leading to variations in photocurrent levels and photoconductive gain.

To investigate the temperature effect of the fabricated UV PDs, a hot plate (BY1010, Dongguan Bangyuan Electronics Co., Ltd.) was placed inside the testing chamber (Figure 6). The previous figure shows the experimental setup, and the UV PD was placed on the platform of the hot plate to heat up the device at constant temperature settings, which started with 40 °C up to 80 °C with an increasing interval of 10 °C. The intensity irradiated on the device was maintained at 6 μWcm^–2^.

The photocurrent responses of both BTO and WS_2_/BTO photodetectors under UV illumination at various temperatures and poling states, as illustrated in Figure 8, offer critical insight into the polarization-dependent behavior of these devices. In both Figure 8a,b, the down-poled state consistently exhibits negative photocurrent values across all temperatures, highlighting a reversal in the current direction due to the inversion of spontaneous polarization. This reversed current, with relatively low magnitude (e.g., ∼1.2 × 10^–6^ A at 40 °C for BTO and ∼2.0 × 10^–6^ A for WS_2_/BTO), remains nearly unchanged across the temperature range up to 80 °C, indicating minimal influence of thermal dipole fluctuation in this state. This stability arises from the weak polarization and suppressed dipole activity, which limits thermal disruption and charge separation efficiency, as also suggested by the negligible change in current density with an increasing temperature.

**Figure 8:**
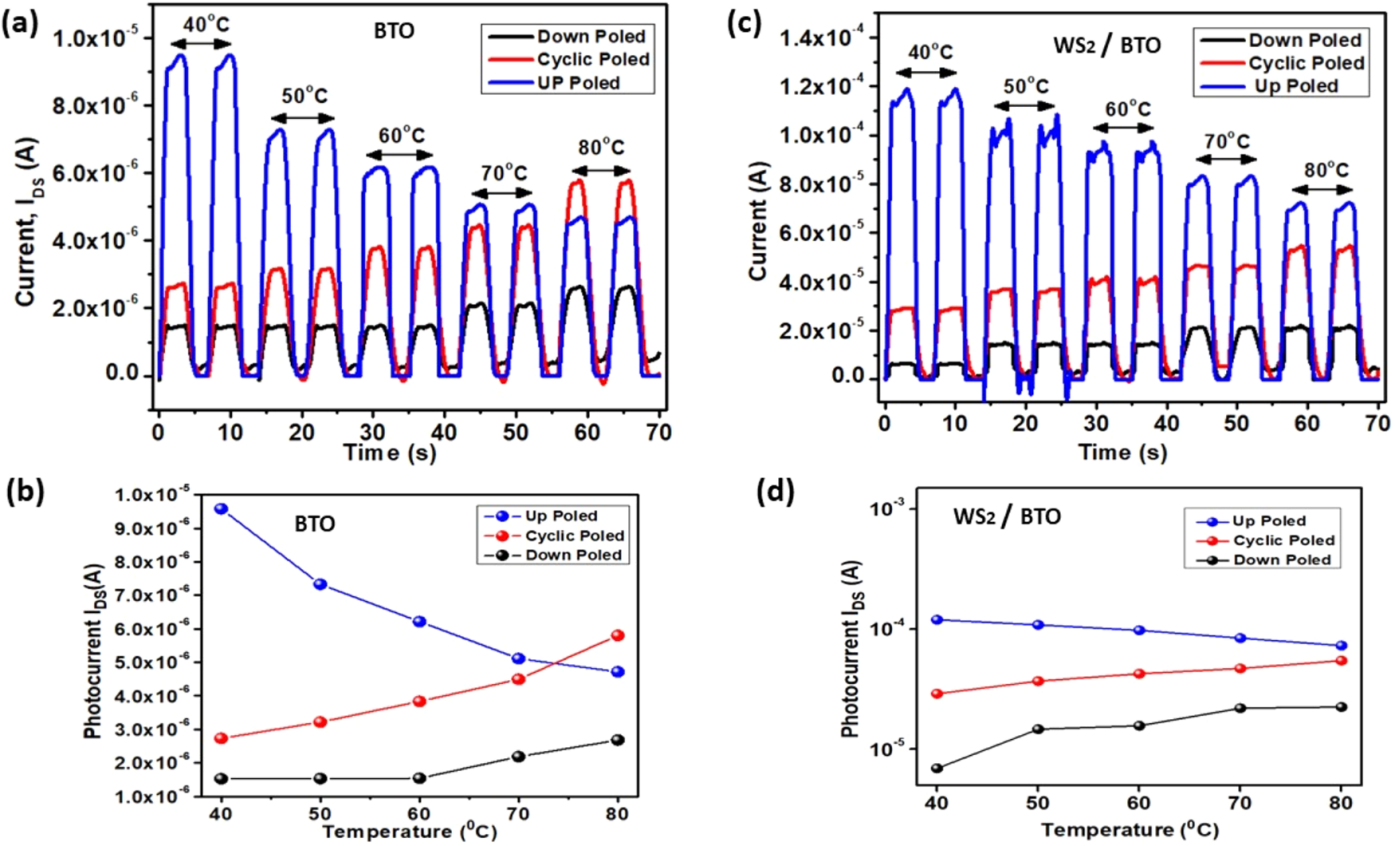
Temperature effect on FeFET, temperature varying from 40 to 80 °C for (a) the BTO device and (b) the WS_2_/BTO device; (c) photocurrent (Ip) of the BTO device and (d) the WS_2_/BTO device.

In contrast, the up poled condition shows the highest photocurrent values at 40 °C (e.g., ∼9.5 × 10^–6^ A for BTO and ∼1.2 × 10^–4^ A for WS_2_/BTO), owing to the strong and stable alignment of ferroelectric dipoles that efficiently aid charge separation and transport. However, as temperature increases, significant dipole agitation leads to reduced polarization strength, resulting in a gradual decrease in photocurrent, dropping to ∼4.2 × 10^–6^ A for BTO and ∼7.0 × 10^–5^ A for WS_2_/BTO at 80 °C. This decline is due to disrupted charge transmission and weakened internal electric fields, which also implies proximity to the ferroelectric Curie point. Additionally, temperature-induced polarization loss reduces the modulation efficiency of the dielectric layer in the field-effect channel, causing threshold shifts and a reduced photoconductive gain.

For the cyclic poled condition, where dipoles are partially randomized, the response behavior is mixed. Initially, an increase in photocurrent is observed with temperature, likely due to thermally assisted dipole alignment enhancing internal polarization. For BTO, the photocurrent increases from ∼2.4 × 10^–6^ A at 40 °C to ∼6 × 10^–6^ A at 80 °C. For WS_2_/BTO, the photocurrent increases steadily from ∼3.0 × 10^–5^ A at 40 °C to ∼5.6 × 10^–5^ A at 80 °C, suggesting that WS_2_ incorporation stabilizes the dipole alignment and suppresses excessive thermal agitation at higher temperatures. This trend suggests the formation of a thermally enhanced depolarization field at intermediate temperatures, which later diminishes due to dipole disorder.

The observed photocurrent variation with temperature is intrinsically linked to the ferroelectric polarization states, which severely influence the carrier transport barrier at the WS_2_/BTO interface. In the up poled condition, the BTO polarization field works to reduce the interfacial barrier height, maximizing the initial current flow at lower temperatures. However, as the temperature increases, the negative effects of phonon scattering (reducing carrier mobility) and enhanced recombination become dominant, leading to the observed decrease in photocurrent.

Conversely, under down poled and cyclic poled conditions, the polarization field initially raises the interfacial barrier, limiting the current flow. As the temperature rises, the energy from thermal activation is sufficient for a significantly larger number of carriers to thermally surmount this high barrier. This activation overcomes the mobility losses, resulting in a net increase in photocurrent with a rising temperature. These opposing temperature-dependent mechanisms explain the distinct trends across the polarization states.

Overall, the WS_2_/BTO hybrid device demonstrates superior photoresponse in all poling states due to enhanced carrier mobility and improved electron–hole pair generation facilitated by the WS_2_ layer. This enhancement is particularly significant in the up poled state, where the peak photocurrent reaches over an order of magnitude higher than that of pure BTO. These results indicate that poling conditions, temperature, and material composition collectively dictate the photocurrent behavior of BTO-based UV photodetectors, with the WS_2_/BTO system offering robust and thermally stable performance.

### 3.7 Further Discussion

The device achieves high efficiency through the synergistic coupling of WS_2_’s strong UV absorption and high carrier mobility with the BTO’s electrostatic control. The ferroelectric field-effect (FeFET) architecture allows for nonvolatile modulation of the WS_2_ channel via the BTO’s remanent polarization, resulting in an ultralow dark current at zero external gate bias (*V*_GS_ = 0). This voltage-free operation, combined with the device’s high responsivity, is crucial for low-power integration and enables the device to function as a compact unit combining sensing and memory. Furthermore, WS_2_ integration reduces the BTO’s coercive field, enhancing the energy efficiency of the required polarization switching.

Reliability is confirmed by the device’s thermal stability across an extended operational range (e.g., 40 to 80 °C), validating its robustness in real-world environments. The device exhibits a large switching ratio and reproducible hysteresis, which is a functional feature that supports stable, nonvolatile memory and switching operations. This structural and electrical integrity ensures consistent, reliable performance under operational conditions.

To position our WS_2_/BTO FeFET against efficient UV photodetectors, we compared it with representative ZnO, GaN, and β-Ga_2_O_3_ devices at near-UV wavelengths. A recent ZnO core/shell device reports ∼4.5 mA W^1–^ at 395 nm,^38^ while textured ZnO networks reach ∼5.85 A W^1–^ at 394 nm under UV illumination.^39^ Ferroelectric-assisted GaN (BaTiO_3_/p-GaN) achieves ∼18 mA W^1–^ at 360 nm at 0 V with fast response (<40 ms), underscoring what self-driven diodes can deliver without gain.^40^ Flexible β-Ga_2_O_3_ nanowire PDs show ∼0.266 A W^1–^ (solar-blind band) with subsecond dynamics,^41^ whereas some gain-dominated architectures report extreme responsivity but at the cost of higher dark current and/or slow recovery.^42^ In this context, our device delivers ∼38.5 A W^1–^ at 375 nm with pA-level dark current at zero gate bias and nonvolatile, polarization-controlled switching over 40–80 °C, which is competitive with (and often above) ZnO and GaN benchmarks at comparable wavelengths and notably adds nonvolatile ferroelectric control that conventional UV photodiodes lack.

The charge carrier transport mechanism in the WS_2_/BTO/TiO_2_ heterostructure is fundamentally governed by the ferroelectric BTO layer, which provides nonvolatile electrostatic control under both dark and illuminated conditions. In the dark, carrier transport is dominated by the electrostatic control exerted by the BTO’s remanent polarization. This polarization establishes a strong internal electric field at the WS_2_/BTO heterojunction, which induces a significant carrier depletion in the WS_2_ channel. This process effectively creates a large Schottky barrier at the interface, severely suppressing the flow of leakage current. The result is an extremely low dark current (∼12.3 pA), which is critical for maximizing the signal-to-noise ratio. Furthermore, the polarization state (i.e., “up” vs “down”) precisely tunes this depletion width, introducing the nonvolatile, memory-like behavior that manifests as observable hysteresis in the device’s *I*–*V* characteristics. The designed band alignment across the entire heterostructure ensures the internal field remains robust, minimizing unwanted conduction.

Upon exposure to UV light, electron–hole pairs are photogenerated primarily within the high-mobility WS_2_ channel and across its interface with the BTO layer. The ferroelectric polarization plays a dual critical role in enhancing the photoresponse. First, the internal field acts as an efficient engine for separating the photogenerated carriers. Second, the polarization induces band bending that facilitates the rapid escape of carriers from the WS_2_, thereby actively suppressing recombination. Electrons are efficiently driven toward the TiO_2_ back layer and collected, while holes are swept out or accumulate at the WS_2_/BTO interface. This highly efficient, polarization-assisted separation mechanism leads to a dramatic increase in the photocurrent (up to 22.5 μA). The resulting stability and efficiency of the photocurrent are dynamically and nonvolatility-modulated by the BTO’s established polarization state, allowing for voltage-free control of the photodetector output.

The device’s performance is characterized by notable figures of merit that confirm its high efficiency and UV selectivity, directly resulting from the ferroelectric gating mechanism. The primary figures of merit demonstrate a high photoconductive gain and low-power operation. The WS_2_/BTO phototransistor achieves a peak responsivity (*R*) of 38.49 A/W at the optimal UV wavelength of λ = 375 nm. This high value is notably achieved under a self-powered condition (*V*_DS_ = 0 V), underscoring the strong internal BTO ferroelectric field’s ability to drive carrier transport without external bias. This responsivity translates to an external quantum efficiency (EQE) of 12727%. An EQE significantly exceeding 100% confirms a substantial photoconductive gain (over 127), which is directly attributed to the efficient carrier separation and amplification provided by the nonvolatile ferroelectric gating mechanism. Furthermore, integrating the WS_2_ layer substantially improves the device’s transient speed. The rise time (τ rise) is improved from 1.2 s (bare BTO) to 0.8 s (WS_2_/BTO), a 33% faster response attributed to the strong, local electric field accelerating carrier collection.

Regarding spectral responsivity, the analysis confirms the device’s inherent UV selectivity. The photoresponse is fundamentally governed by the composite’s absorbance spectrum (Figure 2d in the revision), which exhibits strong absorption at 375 nm, followed by a rapid drop-off toward longer wavelengths. While the WS_2_ component provides a slight extension into the visible range, the minimal overall response in the Vis–IR region validates the device’s effective use as a high-performance, selective UV photodetector.

The WS_2_/BTO device works by a ferroelectric field-effect at the interface. Remanent BTO polarization leaves a bound charge that electrostatically dopes WS_2_ and shifts the metal/WS_2_ barriers. Reversing polarization flips this interfacial charge, giving nonvolatile changes in channel conductance and photocurrent. Under illumination, the built-in field aids carrier separation and lowers recombination. Poling-dependent transfer curves and hysteresis confirm that BTO polarization directly gates WS_2_, not merely a response of BTO alone.

The stability of the WS_2_/BTO phototransistor was evaluated through cycling and environmental tests. Under cyclic bipolar poling (50 Hz, 50 cycles), the P–E loops and *I*–*V*/transfer curves remained consistent, confirming stable polarization switching. Photocurrent was also measured during repeated UV ON/OFF cycles (3.5 s period), where the device maintained a high switching ratio (>10^4^) and a fast rise time (∼0.8 s) over many cycles. In addition, thermal tests from 40–80 °C showed that the WS_2_/BTO device preserved strong, polarization-dependent photocurrent. These results demonstrate a stable device performance under electrical cycling, repeated photoresponse, and elevated temperatures.

Table 1 compares our device to other state-of-the-art photodetectors reported in the literature. The key performance metrics, including operational bias, testing wavelength, and responsivity, clearly highlight the superior performance of our work.

**Table 1:** Comparison of Performance Metrics for our WS_2_/BTO Photodetector with Other Recently Reported WS_2_-Based Heterojunctions

no.	photodetector device	operating voltage (V)	wavelength (nm)	responsivity	elasticity verification	refs
1	WS_2_/porous Si heterojunction	0	625	5.02 mA/W	stiff	^43^
2	WS_2_-anchored MoS_2_	0	580	283 mA/W	stiff	^44^
3	TETA-Gr/WS_2_/LaVO_3_ heterojunction	0	740	0.38 A/W	stiff	^45^
4	GeSe/WS_2_/MoS_2_ van der Waals heterojunctions	0	405	14 mA/W	stiff	^46^
5	back-gated WS_2_/BTO heterostructure	0	375	38.49 A/W	flexible	this work

## 4 Conclusion

Integrating a ferroelectric barium titanate (BTO) dielectric with a monolayer WS_2_ channel produces a UV phototransistor that combines ultrahigh sensitivity, low noise, fast response, nonvolatile switching, and thermal robustness: responsivity jumps from 1.9 A/W (bare BTO) to 38.5 A/W (≈20× enhancement), dark current drops to just 12.3 pA, photocurrent under UV light is amplified from 0.1 to 22.5 μA (≈28× increase), and rise time shrinks from 1.2 to 0.8 s. The device has also been evaluated in various parameters such as DC poling and gate modulation, which results in an increased photocurrent of 1.05 mA at gate modulation with an up poling condition at *V*_GS_ = 2 V for the WS_2_/BTO device. Moreover, ferroelectric poling enables over an order-of-magnitude, voltage-free toggling of photocurrent, while stable performance from 40 to 80 °C demonstrates excellent thermal reliability. Together, these advances overcome the intrinsic absorption and leakage limitations of 2D phototransistors, delivering a low-power, high-performance UV sensor that is ideal for environmental monitoring, wearable health devices, and safety applications.

## Supplementary Material



## Notes

Rietveld refinement plots and fit statistics for BTO-only and WS_2_/BTO/TiO_2_/Ti films (Figures S1 and S2, and Table S1) (PDF)
