# Supplementary material for: Back-Gated WS2‑Enhanced Barium Titanate Phototransistor with Polarization-Controlled UV Responsivity and Enhanced Thermal Stability
Source: ACS Appl Mater Interfaces. 2025 Nov 19;17(48):65715–27. doi: 10.1021/acsami.5c15016 (PMC12679533; doi:10.1021/acsami.5c15016)
Supplement: Supplementary file 1 [file am5c15016_si_001.pdf]

Supplementary Information

Back-Gated WS_2 enhanced Barium Titanate Phototransistor with Polarization-Controlled UV Responsivity and Enhanced Thermal Stability

Rohit Raj Padhi [1], Chiranjit Das [1], Guo-Hua Feng [1,2]*

[1] Institute of NanoEngineering and Microsystems, National Tsing Hua University, Hsinchu 30013, Taiwan

[2] Derpartment of Power Mechanical Engineering, National Tsing Hua University, Hsinchu 30013, Taiwan

*Corresponding Author: Guo-Hua Feng, orcid.org/0000-0003-4322-2255

Email: ghfeng@pme.nthu.edu.tw

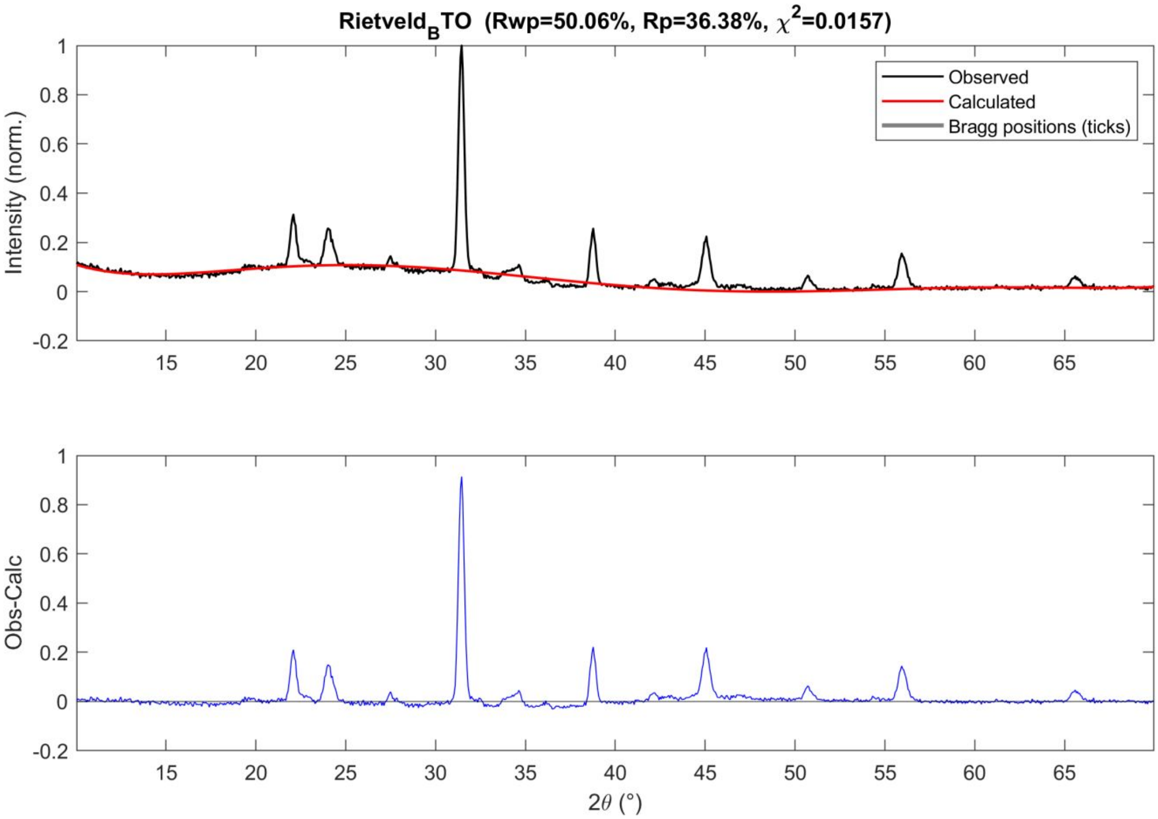

Fig. S1. Rietveld refinement of the hydrothermally grown BTO/TiO_2/Ti film in the 10–70° 2θ range. Refinement parameters: Rwp = 50.06 %, Rp = 36.38 %, χ^2 = 0.01572, zero = 0.0060°. The diffraction pattern is well modeled using tetragonal $BaTiO_3$ (P4mm) together with TiO_2 (anatase ± rutile). The refined lattice constants of the BTO phase are a = 4.0045 Å, c = 4.2006 Å (c/a = 1.04899), confirming the expected tetragonal distortion. The difference trace is featureless across the scan, and all observed peaks are reproduced without any unidentified reflections. The relatively high Rwp value arises from substrate scattering and preferred orientation typical of thin-film measurements.

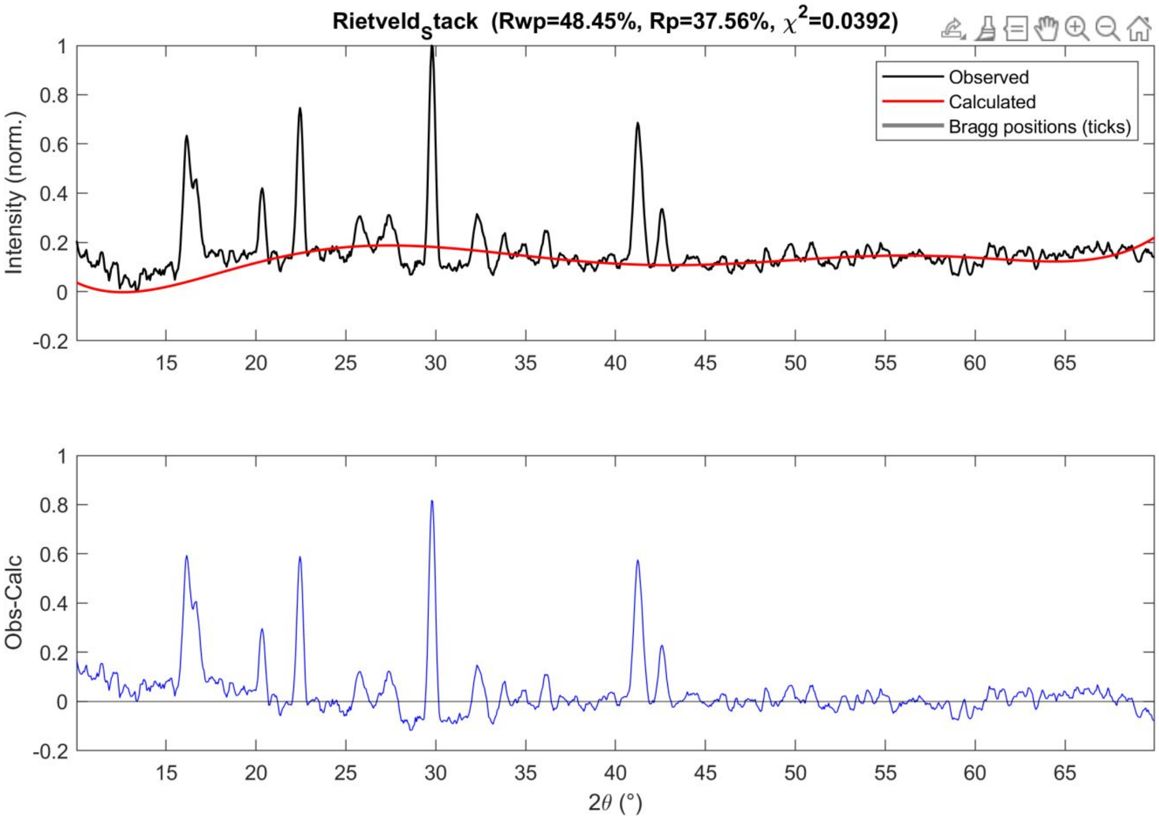

Fig. S2. Rietveld refinement of the WS_2/BTO/TiO_2/Ti stack in the 10–70° 2θ range. Refinement parameters: Rwp = 48.45 %, Rp = 37.56 %, χ² = 0.03925, zero = 0.0063°. The model includes 2H-WS_2 + t-$BaTiO_3$ + TiO_2 (anatase ± rutile), successfully accounting for all Bragg reflections. The BTO lattice constants remain a = 4.0045 Å, c = 4.2006 Å (c/a = 1.04899), demonstrating that the WS_2 integration does not alter the ferroelectric tetragonal structure. No secondary crystalline phases above ~2–3 % are detected within the instrumental sensitivity. The smooth residual curve confirms a statistically consistent fit despite the polycrystalline thin-film texture.

Table S1. Refinement summary

Pattern	Rwp (%)	Rp (%)	χ²	zero (°)	U	V	W	η	a (Å)	c (Å)	c/a
BTO-only	50.06	36.38	0.01572	0.0060	0.0200	0.0100	0.0300	0.550	4.0045	4.2006	1.04899
WS_2/BTO stack	48.45	37.56	0.03925	0.0063	0.0200	0.0100	0.0300	0.550	4.0045	4.2006	1.04899

Note: Phase fractions derived from scale factors are semi-quantitative due to thin-film absorption and texture effects. All observable Bragg intensity in 10–70° 2θ is modeled by the listed phases, with no unidentified crystalline residues.
